# Role of Risk Assessment in Prevention of Work-Related Accidents and Diseases in Hospital Staff

**DOI:** 10.25122/jml-2020-0048

**Published:** 2020

**Authors:** Dan Ioan Boariu, Petru Armean

**Affiliations:** 1. Occupational Safety and Health Department, Bucharest Emergency University Hospital, Bucharest, Romania; 2. “Carol Davila” University of Medicine and Pharmacy Bucharest, Bucharest, Romania

**Keywords:** Risk assessment, work accident, morbidity, professional diseases, healthcare professionals, AC – air conditioning, ARACT - Martinique Regional Association for the Improvement of Working Conditions, d – days, DCT – diagnostique des conditions du travail, DSF - diagnostic safety form, FMEA – failure mode, effects and criticality analysis, FTA - fault tree analysis method, GRL – global risk level, Hazop – hazard-operability, HR – human resources, ICU – intensive care unit, IERCM – Bucharest Institute of Work Capacity Expertise, INCDPM - National Research and Development Institute for Labor Protection Bucharest, INVALID 1st – first degree invalidity MORT - management oversight and risk tree, LEST – Laboratoire d’ergonomie et de sociologie du travail, MLSSF – Ministry of Labor, Social Solidarity and Family, MOSAR - Method Organized Systematic Analysis of Risk, OR – operation room, OSH - occupational safety and health, PRA – preliminary risk analysis, PRL – partial risk level, PUO – performing unforeseen operations through work tasks, SDQ - safety diagnosis questionnaire, SME - small and medium-sized enterprises, SOBANE - screening, observation, analysis, expertise, TIW – temporary incapacity for work

## Abstract

Risk assessment is an essential component of the occupational health and safety event prevention activity.The purpose of this study is to choose the most appropriate risk assessment method for hospitals. The main methods were compared. There are many assessment methods, each with its advantages and disadvantages, but none has been adapted to the specificities of hospital activity. We adapted the workplace assessment sheet from the INCDPM (National Research and Development Institute for Labor Protection Bucharest) method to the specific of the hospital units and used this method at the level of jobs, within the hospital’s departments, calculating the global risk level per job position, workplace (department), and hospital. The clinical departments global risk level exceeds the average (3.00) for all jobs, but does not exceed, however, 3.50, representing an acceptable security level. For assess the psychosocial risks we used the ELVIE method. Looking ahead, the methods should be adapted to allow both numerical presentation of the results and graphic.

## Introduction

### Purpose of the research

The starting point in the prevention of accidents at work and occupational illnesses is the assessment of risks in the work system. Whether it is a job, a department, or a hospital, this analysis allows risk-ranking according to their size and the efficient allocation of resources for priority measures.

In order to fulfill the preventive measures provided under the Occupational health and safety (OSH) Law number 319/2006, which transposes the provisions under Directive 89/391/EEC into the national legislation, the employer is obligated to draft a prevention and protection plan, based on the risk assessment. Individual OSH instructions shall be drawn up based on this plan [1].

### Risk assessment methods

Risk is the relationship between the likelihood of an event occurring and the severity of the possible consequences: work accident, occupational or occupational-related disease. The risk assessment involves identifying all risk factors in the work system (composed of work equipment, working environment, work task, and worker) and quantifying their size based on the combination of the two parameters (severity and frequency of the maximum possible consequence on the human body), thus achieving partial risk levels (PRL) for each risk factor, i.e., overall (global) risk levels (GRL) for the whole system analyzed (workplace). This principle is the basis for the different methods of practical application. Assessing a state requires finding a correlation between the state and an indicator that can be assigned or calculate its dimension (the assessment of which can be performed directly or through the opposite state) [2].

Risk assessment methods are numerous, but there are only two assessment principles – post-accident/illness – ‘a posteriori’ methods (assessment criteria are the number of events occurring and their severity) and pre-accident/illness (criteria are the likelihood and potential severity of events that could occur) - ‘a priori’ methods. A posteriori assessment uses accident or occupational illness morbidity rates (indices). Absolute indicators (number of accidents, number of days of incapacity, and others) allow the overall characterization of the safety situation. However, they do not allow for comparisons, unlike the relative indicators (frequency index, severity index, and others). This assessment does not consider potential risk situations, to ascertain facts already occurred and not the prevention. A priori assessment shall consider the possibilities of events occurring in a system with predictive and preventive value. It is based on the direct and complete identification of risks, allowing their quantification and ranking, with a view to prioritizing preventive measures [3].

A priori methods:

1. Controls and verifications (specialized inspections, limited to the activity with the dominant risk). These do not consider the risks concerning work tasks and workers, and the analysis is purely qualitative [3].

2. Analytical methods

a. Heinrich model-based methods: the accident is viewed as a result of a chain of risks of a technical (hazardous conditions) and human nature (hazardous actions), with emphasis on the latter. These use a positive behavior (challenging to quantify) as a reference model, and the assessment is qualitative [4].
b. Systems reliability theory-based methods are built on a ‘deficiency rate’, based on inductive and deductive reasoning, which allows for the detection of system malfunctions: FMEA (failure mode, effects and criticality analysis), FTA (fault tree analysis method), PRA (preliminary risk analysis), ‘what – if’ method, MOSAR (Method Organized Systematic Analysis of Risk), Delphi technique. These methods limit themselves to the technical factor, without considering the work environment, the task, and the worker; they imply a considerable work volume and are expensive [4, 5].
c. Systems ergonomics based methods: Hazop (hazard – operability), DSF (diagnostic safety form), DCT (“diagnostique des conditions de travail”, french for “diagnosis of working conditions”), SDQ (safety diagnosis questionnaire), MORT (management oversight and risk tree), IERCM (Bucharest Institute of Work Capacity Expertise), Renault, LEST (“Laboratoire d’ergonomie et de sociologie du travail”, french for “Ergonomics and sociology of work laboratory”). These methods also present disadvantages: HAZOP may be applied only to automated processes, DSF is based exclusively on the opinions of the workers, DCT has only a sociological approach, IERCM refers solely to getting sick, and not to accidents, Renault focuses on ergonomic organization, and LEST on fatigue [5].

Some methods address distinct categories of risks (chemical, biological, psychosocial) or elements of the work system (electrical work equipment, control systems). 

### The INCDPM method of occupational accident and disease risk assessment

The Ministry of Labor, Social Solidarity and Family (MLSSF) has approved a single method for assessing these risks, developed within the National Research and Development Institute for Labor Protection (INCDPM) from Bucharest. It is part of the analytical methods aimed at quantitatively determining the risk level, based on systemic analysis and assessment of occupational injury and illness risks. The principle consists in identifying all risk factors in the system (pre-established checklists) and quantifying the size of the risk (the combination of severity and frequency of the maximum foreseeable consequence). The presence of risk factors determines the existence of risk in a system. Consequently, in order to assess the risk, the following steps need to be taken: defining the system to be analyzed (workplace) – identifying the risk factors in the system – assessing the risks (establishing the consequences of the action, therefore determining the severity) and establishing the probability of action on the worker – assigning the risk levels depending on the severity and probability of the consequences of the action of the risk factors – ranking the risks and establishing the prevention priorities – proposing preventive measures [6].

The necessary stages for assessing occupational safety within a system, previously described, are attained using the work instruments, presented below. The “Identification list of risk factors” comprises the primary categories of risk factors, grouped according to the criteria of the generating element within the system. The “List of possible consequences” of the action exerted by risk factors on the human body is meant to aid in applying the rating scale of severe consequences. It comprises the categories of lesions and injuries, possible localization of the consequences concerning the body’s anatomic-functional structure, and the generic severity of the consequence, from minimal severity (small lesions) to maximum severity (death). The “Rating scale of the severity and probability” of consequences of the actions exerted by the risk factors on the human body is a consequence classification grid, which groups them in 7 severity classes and 6 occurrence probability classes. The section that refers to the severity of consequences is based on the medical criteria of clinical, functional diagnosis, and the assessment of work capacity elaborated by the Ministry of Health and the MLSSF. The grid of the probability classes contains 6 frequency classes - from once every over 10 years to once every less than a month. The expression of the existing risks within the analyzed system is done with the aid of the risk assessment grid, and it is done in the shape of a severity - occurrence frequency pair, leading to PRL (for each risk factor). The Occupational Risk/security level classification scale is used in appreciating the expected risk level and the expected security level, respectively (from 1 to 7). The Workplace assessment sheet is the centralizing document of all occupational accident and/or sickness risks identification and assessment operations. This form comprises workplace identification data: unit, department, workplace; assessor identification data; generic components of the workplace; nomination of identified risk factors; the concrete manifestation forms of identified risk factors (description, parameters, and functional features); the maximum predictable consequence of the action of risk factors; the expected severity and probability class; the risk level. The “Sheet of proposed measures” is a form for the centralization of prevention measures that need to be applied and the results of the workplace’s assessment.

The workplace assessment sheet, which includes the GRL per workplace, forms the basis for the schedule to prevent accidents at work and occupational illnesses.

Going forward from the PRL’s, we calculate the GRL’s by position, department, and hospital [6].

### ELVIE Occupational Psychosocial Risk Assessment Method

The ELVIE questionnaire, designed by the Martinique Regional Association for the Improvement of Working Conditions, contains 144 questions, with multiple choices (disagree, agree, not known, and so on), grouped into 15 categories: 1. Assessing the work performed, 2. Employment relations 3. Autonomy 4. Provisions 5. Meaning of work 6. Prospects 7. Workload 8. Hygiene, security, material conditions 9. Contribution, retribution 10. Interest, diversity of work 11. Trust, cooperation 12. Labor splitting 13. Polyvalence 14. Communication, briefing 15. Skills appropriate to work. The questionnaire answers highlight poorly managed tensions (psychosocial risks, complaints) using color coding (red – poorly managed tensions, yellow – potential tensions, green - balance) [7].

### Self-assessment of security at the level of small and medium-sized enterprises (SME’s)

The method contains 119 open questions, assuming a score from 0 to 5, allowing for both qualitative (strengths – weaknesses) and quantitative (percentages) assessments [8].

### SOBANE Strategy and the Deparis Guide

The SOBANE strategy aims at solving the coordination problems of OHS professionals, organizing the collaboration as effectively and economically as possible to efficiently prevent occupational risks. It has four progressive levels of intervention: screening (identification) - observation - analysis – expertise. The Deparis Guide helps to find immediate solutions to problems related to working conditions, using the SOBANE strategy. Essentially, during a routine visit (screening), a problem is examined in detail (observation); a prevention counselor is requested (OSH officer, occupational physician) for analysis, and in extreme cases, an expert is asked (toxicologist, organizational psychologist). The guide comprises 18 tables, which address different work situations (organization, security, ergonomics, environment, psycho-organizational situations). Following the group discussion, the leader assigns each problem a color: red (unsatisfactory conditions, needs improvement), orange (relatively satisfactory conditions, can be improved), green (satisfactory conditions). Subsequently, the summary is submitted to the management. The guide allows for the inventory of all aspects related to working conditions, the substantiation of immediate and pertinent solutions, and the determination of priority issues, which need to be deepened [9].

## Material and Methods

### Adoption of the INCDPM method within the health care sector

Given the advantages and disadvantages of the methods mentioned above, we can observe that the most comprehensive method is INCDPM, as it refers to all four components of the labor system. We believed that it is an adequate assessment method for risks in hospitals if it is improved.

With this purpose, we adapted the risk factor identification list and the workplace assessment sheet to the specific of hospitals: we developed and customized the sections of chemical risks (biocide substances, reactive agents, paints), biological risks (parasite infestation, human aggressions), and other specific risks (radiation). With the purpose of adapting the workplace assessment sheet to the specific of the hospitals, we studied the specialized literature (and ascertained that the issue of risk assessment is not approached in a unitary manner in the same country, being approached depending on the basic professional training of the assessors - physicians, engineers, psychologists: in most cases, data being available only from the perspective of the OHS engineer or from the perspective of the occupational medicine physician); we visited hospitals and studied the conclusions of the occupational medicine exams, as well as the risk-assessment works. 

### Applying the ELVIE method

We used the questionnaire, which we applied anonymously to personnel that has been working for at least one year in that respective position.

## Results and Discussions

### Assessment through the INCDPM method

We applied the method at the level of jobs (occupations, professions) within the hospital’s departments, calculating the GRL per job position, workplace (department), and hospital.

To exemplify, we provided the job position assessment sheet for physicians in the surgery department in Appendix 1.

The following results were obtained at the level of the surgical departments (Table 1).

**Table 1:** Assessed risk levels for surgical departments.

Item no.	Category	Risk level
**1**	Surgery department physicians	3.27
**2**	Surgery department medical nurse	3.21
**3**	Nurse, caregiver	3.16
**4**	Stretcher-bearer	3.08
**5**	Medical registrar / PC operator	3.12

The global risk level was 3.19 for the surgery department and we also calculated GRL for the clinical departments, the results being presented in Table 2. The global risk level was 3.22 for clinical departments. However, the risk level exceeded 3.00 in the case of all jobs. In the case of certain jobs, the value even exceeds 3.30. The GRL (3.22) does not exceed, however, 3.50, representing an acceptable security level.

**Table 2:** Global risk levels for clinical departments.

Item no.	Department	Risk level
**1**	Medical departments	3.14
**2**	Surgical departments	3.19
**3**	ICU	3.33

### Assessment of psychosocial risks through the ELVIE method

The questionnaire was applied to 50 employees in all professional categories involved in medical assistance. The analysis of the answers indicates that there are poorly managed tensions (discontent) regarding the following issues: retribution, work fractioning, tasks, hygiene conditions, security and work materials, provisions and autonomy in the workplace (6 groups out of 15).

The distribution by professional categories has emphasized that the poorly managed situations refer to the following issue groups: while medical nurses complain especially about issues regarding perspective, work diversity, trust, and cooperation, communication, information and competence, the lack of appreciation for their work, physicians complain especially about issues regarding contradictory provisions and the work tasks. All categories complain to the same extent about issues related to hygiene, security, retribution, and work fractioning.

## Conclusions

Hospital staff is exposed to a combination of occupational hazards, covering almost the entire spectrum of risks. In order to adapt the list of risk factors identification and the job description to the specifics of the hospitals, in addition to the risks included in the identification list and the workplace assessment sheet by the authors of the INCDPM evaluation method, risks must be included and customized according to the specifics of the different sectors within the hospitals (medical wards, surgical wards, anesthesia, ICU, medical tests laboratory, radiology, outpatient consultation practices, food facility, functional structures – human resources, accounting, and so on). The focus should be on specific risks: ionizing radiation, electromagnetic fields, microbiological agents in the hospital environment, psychosocial risks, poor workload design (including due to staff shortages).

The INCDPM method can be adapted for the health sector, and used efficiently, with the following conditions:

• The lead assessor is an occupational physician with a thorough knowledge of OSH (graduation of risk assessment courses);
• The medical staff, with a thorough knowledge of the specifics of the assessed sector, should be involved in the assessment team.

Given that the INCDPM method is sufficient for chemical risk assessment and that the maximum risk should be taken into account for biological agents (given that, on the one hand, patients infected with biological agents of all types may come to the emergency hospital and, on the other hand, apparently healthy people but potential carriers of dangerous agents may be referred to some services), psychosocial risks are to be assessed separately (for example using the ELVIE method).

Each of the presented methods has specific advantages (the INCDPM method provides quantitative, accurate data, and the ELVIE and SOBANE methods allow graphical presentation, being easier to understand by the executive and management staff). Looking ahead, the methods should be adapted to allow both numerical and graphic representations of the results (like the method of self-assessment for SMEs).

## Conflict of Interest

The authors declare that there is no conflict of interest.

## Appendix 1

**Table:**

**UNIT:**	**WORKPLACE ASSESSMENT SHEET**	**Number of persons exposed:**			
**Department: Surgery 1**		**Exposure duration: hours / day**			
**Job position: Physician**		**Assessment team:**			
**Labour system component**	**Identified risk factors**	**Concrete manifestation forms of identified risk factors (description, parameters)**	**Maximum predictable consequence**	**Severity class**	**Frequency class**	**Partial risk level**
**Work equipment**	**Mechanic risk factors**	Auto-triggers or auto-blockages of fluids: oxygen	DEATH	7	1	3
		Movement under the effect of gravity: slipping, rolling, (free) fall, free leak; spill, slid: (objects on) furniture, stretcher, carriages	TIW 46-180 d	3	3	3
		Movement under the effect of propulsion, projection of particles: broken light bulb, window shards, monitor fragments	INVALID. 1st	6	2	4
		Dangerous surfaces: needles, scissors, stapler, furniture edges	TIW 3 - 45 d	2	5	3
		Recipients under pressure (O.R. oxygen)	DEATH	7	1	3
	**Thermal risk**	Flames, sparks: short-circuit (ICU monitor)	TIW 46-180 d	3	3	3
	**Electrical risk**	Direct contact: conductors with deteriorated isolation	DEATH	7	1	3
		Indirect contact: deterioration of protection circuit	DEATH	7	1	3
	**Chemical risk**	Allergens: talcum, latex gloves etc	TIW 46-180 d	3	5	4
	**Biological risk**	Biological products: blood, faeces, sputum, wound secretions	DEATH	7	2	4
**Work environ-ment**	**Mechanic risks**	Natural calamities (storm, flood, landslides, earthquakes etc.).	DEATH	7	1	3
	**Physical risk factors**	Inadequate air temperature: air conditioning (AC) installation positioned inappropriately, impossibility of being adjusted	TIW 3 - 45 d	2	5	3
		Air currents: AC installations inadequately adjusted or positioned and/or opened windows (the AC is not working)	TIW 46-180 d	3	6	4
		Air ionization	TIW 3 - 45 d	2	6	3
		Noise (interferes with concentration, leading to over-solicitation)	TIW 3 - 45 d	2	6	3
		diminished lighting level; excessive artificial lighting (stress)	TIW 3 - 45 d	2	6	3
		Brightness: reflection (monitor), windows (visual fatigue)	TIW 3 - 45 d	2	6	3
		Intermittent lighting: neon tube	TIW 3 - 45 d	2	6	3
		non-ionized radiation: UV lamp	INVALID. 1st	6	2	4
		Ionized radiation (X rays): intraoperative x-rays	DEATH	7	2	4
		Electrostatic charging during prolonged surgical interventions	TIW 46-180 d	3	5	4
		Powder in suspension: dust, talcum (allergies)	TIW 3 - 45 d	2	5	3
	**Chemical risk factors**	Gases, vapors, toxic aerosols; disinfectants, anaesthetics	TIW 46-180 d	3	5	4
		Inflammable gases: oxygen, nitrogen protoxide (OR)	DEATH	7	2	4
		Substances that irritate the airways and the eyes (surfaces disinfectants: peracetic acid, quaternary ammonium compounds)	TIW 3 - 45 d	2	6	3
		Substances that cause skin allergies (disinfectant for operating field or for hands: chlorhexidine)	TIW 46-180 d	3	5	4
		Substances that irritate the airways and the eyes (disinfectants: ethanol, biphenyl, ammonium)	TIW 3 - 45 d	2	6	3
		Substances that irritate the airways (ethanol): hand disinfectant	TIW 3 - 45 d	2	6	3
		Substances that irritate the airways, eyes, cause allergies (endoscopy disinfectants: glutaraldehyde, peracetic acid).	TIW 3 - 45 d	2	4	2
	**Biological risk factors**	Microorganisms: Koch bacilli, Pseudomonas, hepatitis viruses, fungi: recipients may be dirty with biological products, thus risking contamination of surfaces, of personnel, documents etc	DEATH	7	2	4
		Parasites: fleas, lice, scabies, helminths	TIW 3 - 45 d	2	5	3
		Dangerous animals (bees, wasps etc): anaphylactic shock	DEATH	7	1	3
	Psychosocial environment	Physical aggression (agitated patients)	TIW 3 - 45 d	2	6	3
		Verbal aggression: patients, relatives	TIW 3 - 45 d	2	6	3
		Tense relations within the collective/with the patients (inadequate relationships: wrongs between colleagues etc)	TIW 3 - 45 d	2	5	3
**Work task**	**Inadequate content of work task in relation to security requirements**	Lack of satisfaction in work: confrontation with death, pain	TIW 3 - 45 d	2	5	3
		Frequent hand washing	TIW 3 - 45 d	2	6	3
		Wrong rules, operations, processes: omission to verify earthing	DEATH	7	1	3
		Absence of certain operations	DEATH	7	1	3
		Erroneous succession of operations	DEATH	7	1	3
		Static effort: prolonged orthostatism	TIW 46-180 d	3	5	4
		Forced or vicious work positions	TIW 3 - 45 d	2	5	3
		Great work rhythm: a large number of patients	TIW 3 - 45 d	2	6	3
		Constant demand for attention (neuroses)	TIW 3 - 45 d	2	6	3
		Difficult decisions in a short period of time	TIW 3 - 45 d	2	6	3
		Short cycle or extremely complex repetitive operations	TIW 3 - 45 d	2	6	3
**Worker**	**Wrong actions - carrying our unpredicted operations within the work task**	Inefficient execution of operations: removing cables from outlets with wet hands, errors in manipulating contaminated object etc.	DEATH	7	1	3
		Wrongful use of protection means	DEATH	7	1	3
		Non-synchronization of operations (delays/acceleration): closing/opening doors (getting fingers stuck); surgical gestures	TIW 3 - 45 d	2	6	3
		Performing unforeseen operations through work tasks (PUO): electrical grid repairs, replacement of fuses, supply cables etc.	DEATH	7	1	3
		PUO: interruption of electricity	DEATH	7	1	3
		PUO: stationing in dangerous areas: electrical panel, auto-access areas; Smoking in fire risk areas (high thermal density: archive)	DEATH	7	1	3
		PUO: movement with same-level fall hazard: slipping; stumbling	TIW 3 - 45 d	2	5	3
		PUO: movement with a hazard of falling from heights, through imbalance; slipping; rushing, lack of attention, negligence	TIW 46-180 d	3	5	4
		Accident-prone communications	DEATH	7	1	3
		Incorrect adjustment of display, lighting features (visual fatigue)	TIW 3 - 45 d	2	5	3
		Positioning of equipment without respecting ergonomic rules	TIW 46-180 d	3	4	3
		Momentary factors: fatigue, disease, emotions, depression, professional / family conflicts, food factors, voluntary efforts	DEATH	7	1	3
		Work under the influence (alcohol), in advanced state of fatigue	DEATH	7	1	3
	**Omissions**	Not respecting the hygiene, security traffic rules etc	DEATH	7	1	3
		Not using protection means (outlets with earthing, protection etc)	DEATH	7	1	3

Note: Global risk level (GRL) = 3.27.

## References

[1] *** Occupational Health and Safety Law 319/2006 (2006).

[2] Darabonț Al., Pece Șt., Dăscălescu A (2001). – Managementul Securității și Sănătății în Muncă.

[3] Darabonț Al., Pece Șt., Dăscălescu A (2001). – Managementul Securității și Sănătății în Muncă.

[4] Darabonț Al., Pece Șt., Dăscălescu A (2001). – Managementul Securității și Sănătății în Muncă.

[5] Darabonț Al., Pece Șt., Dăscălescu A (2001). – Managementul Securității și Sănătății în Muncă.

[6] INCDPM (2003). Metodă de evaluare a riscurilor de accidentare și îmbolnăvire profesională la locurile de muncă.

[7] Laport Danielle (2009). ‘ELVIE, o metodă de diagnostic și de prevenire a riscurilor psihosociale în muncă’.

[8] Darabonț Al., Nisipeanu S., Darabonț D. (2002). Auditul Securitatății și sănătății în muncă.

[9] Malchaire J., Naghi E. (2014). La strategie SOBANE et le guide Deparis pour la gestation participative des risques professionnels. Romanian Journal of Occupational Medicine.

